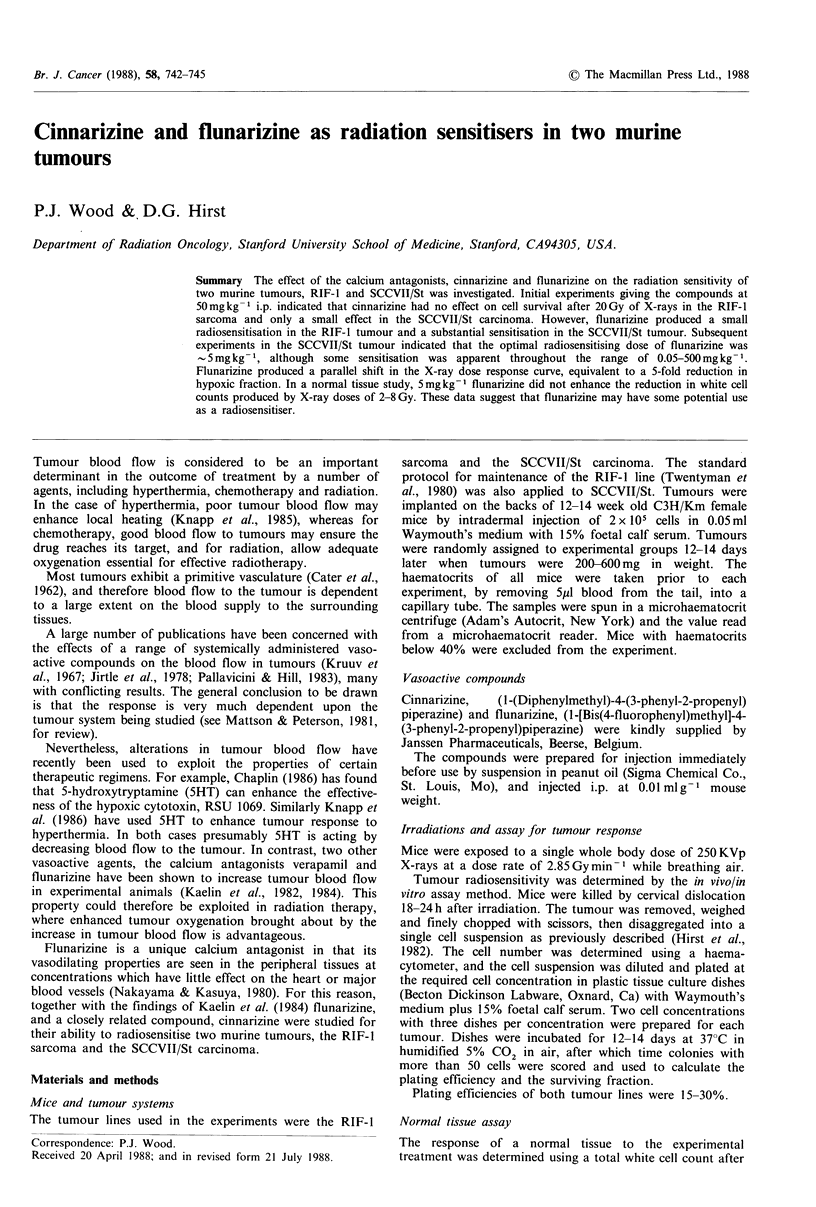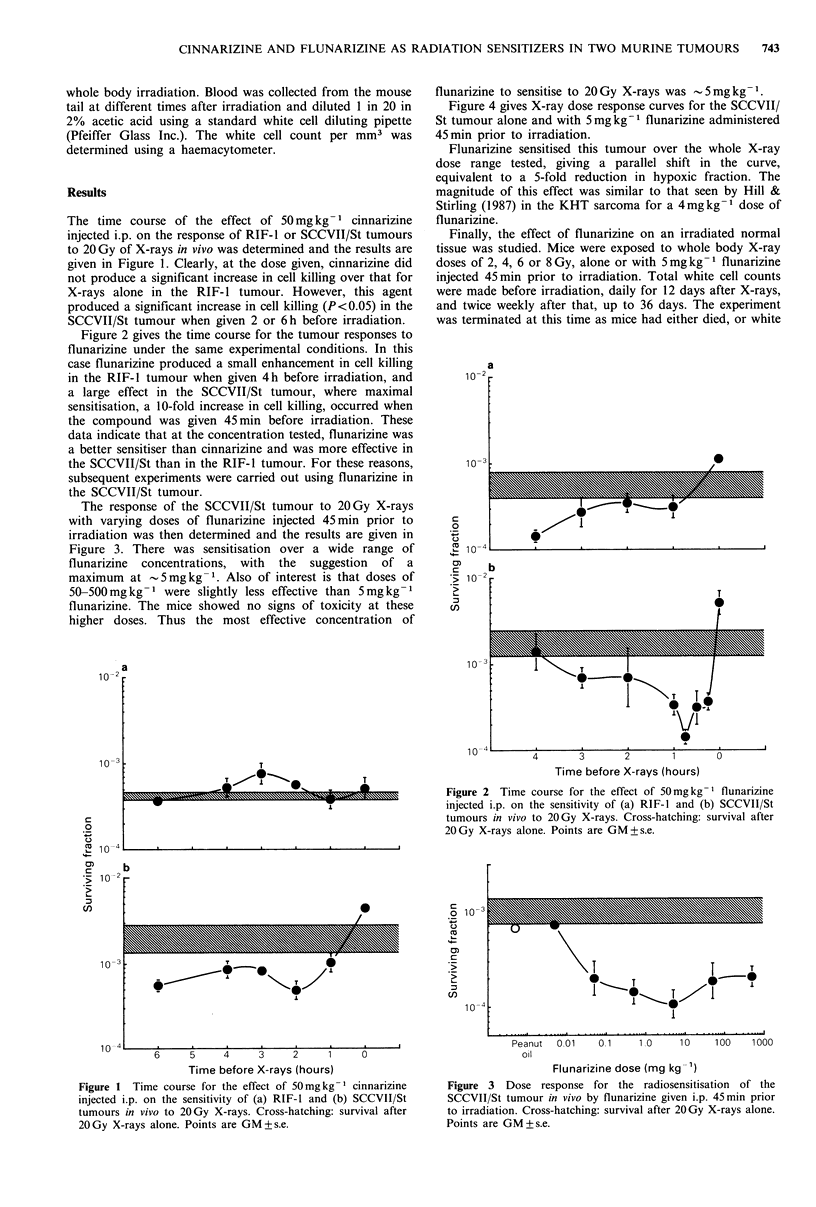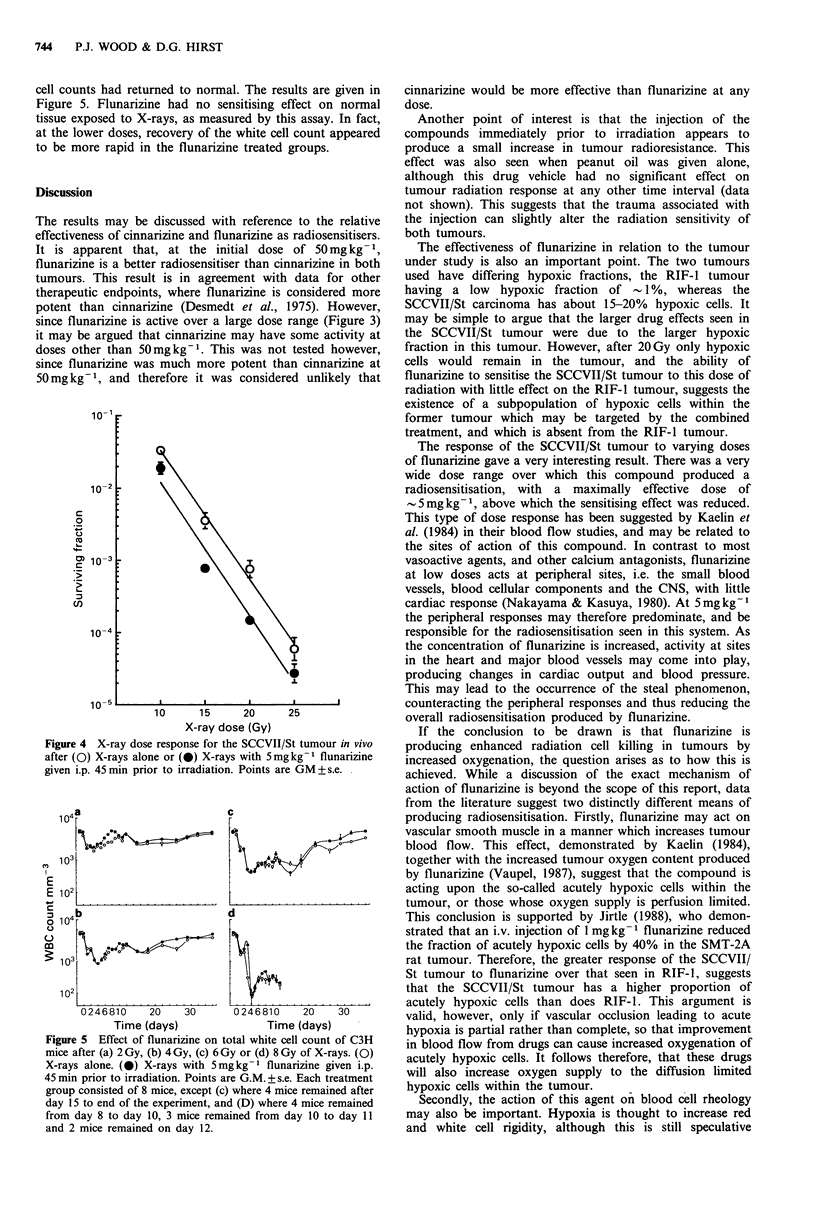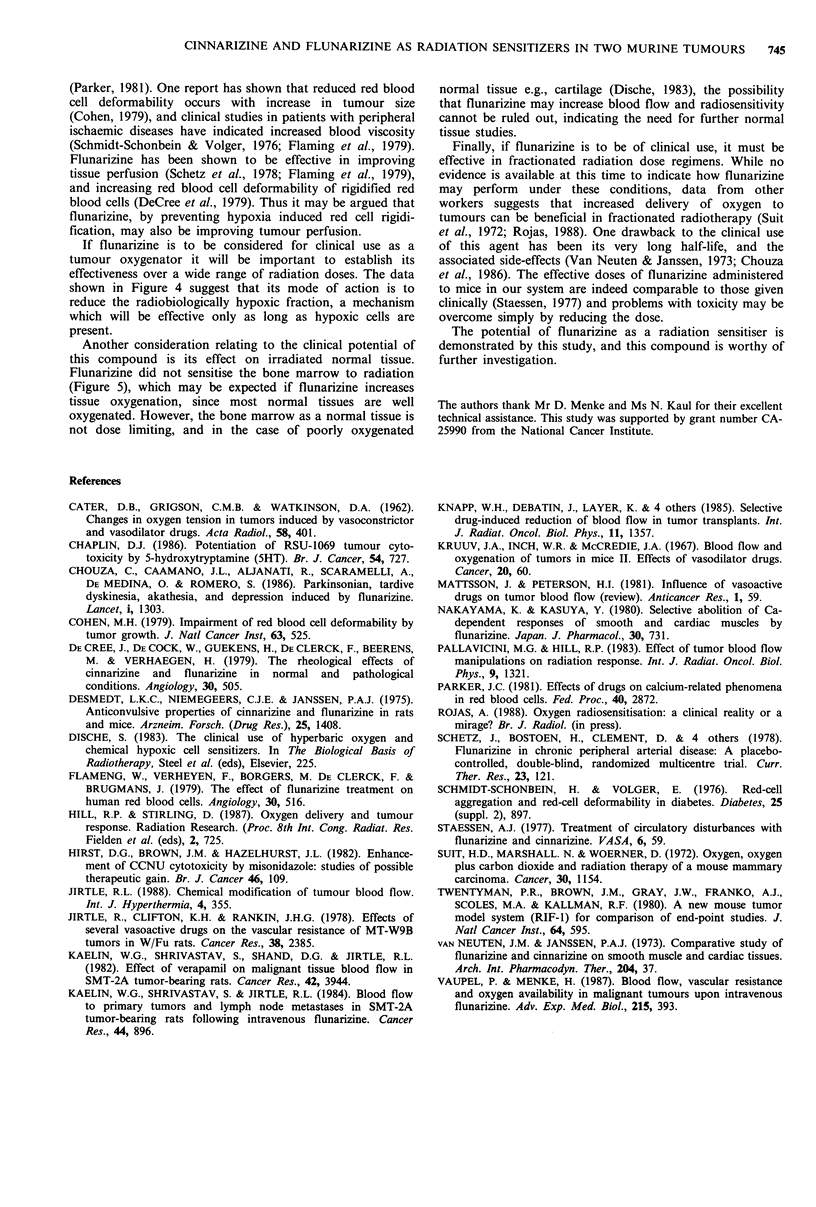# Cinnarizine and flunarizine as radiation sensitisers in two murine tumours.

**DOI:** 10.1038/bjc.1988.301

**Published:** 1988-12

**Authors:** P. J. Wood, D. G. Hirst

**Affiliations:** Department of Radiation Oncology, Stanford University School of Medicine, CA 94305.

## Abstract

The effect of the calcium antagonists, cinnarizine and flunarizine on the radiation sensitivity of two murine tumours, RIF-1 and SCCVII/St was investigated. Initial experiments giving the compounds at 50 mg kg-1 i.p. indicated that cinnarizine had no effect on cell survival after 20 Gy of X-rays in the RIF-1 sarcoma and only a small effect in the SCCVII/St carcinoma. However, flunarizine produced a small radiosensitisation in the RIF-1 tumour and a substantial sensitisation in the SCCVII/St tumour. Subsequent experiments in the SCCVII/St tumour indicated that the optimal radiosensitising dose of flunarizine was approximately 5 mg kg-1, although some sensitisation was apparent throughout the range of 0.05-500 mg kg-1. Flunarizine produced a parallel shift in the X-ray dose response curve, equivalent to a 5-fold reduction in hypoxic fraction. In a normal tissue study, 5 mg kg-1 flunarizine did not enhance the reduction in white cell counts produced by X-ray doses of 2-8 Gy. These data suggest that flunarizine may have some potential use as a radiosensitiser.


*Br. J. Cancer* (1988), **58**, 742–745 © The Macmillan Press Ltd., 1988

### Cinnarizine and flunarizine as radiation sensitisers in two murine tumours

P.J. Wood & D.G. Hirst

*Department of Radiation Oncology, Stanford University School of Medicine, Stanford, CA94305, USA.*

**Summary** The effect of the calcium antagonists, cinnarizine and flunarizine on the radiation sensitivity of two murine tumours, RIF-1 and SCCVII/St was investigated. Initial experiments giving the compounds at $50\,mg\,kg^{-1}$ i.p. indicated that cinnarizine had no effect on cell survival after 20 Gy of X-rays in the RIF-1 sarcoma and only a small effect in the SCCVII/St carcinoma. However, flunarizine produced a small radiosensitisation in the RIF-1 tumour and a substantial sensitisation in the SCCVII/St tumour. Subsequent experiments in the SCCVII/St tumour indicated that the optimal radiosensitising dose of flunarizine was $\sim 5\,mg\,kg^{-1}$, although some sensitisation was apparent throughout the range of $0.05–500\,mg\,kg^{-1}$. Flunarizine produced a parallel shift in the X-ray dose response curve, equivalent to a 5-fold reduction in hypoxic fraction. In a normal tissue study, $5\,mg\,kg^{-1}$ flunarizine did not enhance the reduction in white cell counts produced by X-ray doses of 2–8 Gy. These data suggest that flunarizine may have some potential use as a radiosensitiser.

Tumour blood flow is considered to be an important determinant in the outcome of treatment by a number of agents, including hyperthermia, chemotherapy and radiation. In the case of hyperthermia, poor tumour blood flow may enhance local heating (Knapp *et al.*, 1985), whereas for chemotherapy, good blood flow to tumours may ensure the drug reaches its target, and for radiation, allow adequate oxygenation essential for effective radiotherapy.

Most tumours exhibit a primitive vasculature (Cater *et al.*, 1962), and therefore blood flow to the tumour is dependent to a large extent on the blood supply to the surrounding tissues.

A large number of publications have been concerned with the effects of a range of systemically administered vasoactive compounds on the blood flow in tumours (Kruuv *et al.*, 1967; Jirtle *et al.*, 1978; Pallavicini & Hill, 1983), many with conflicting results. The general conclusion to be drawn is that the response is very much dependent upon the tumour system being studied (see Mattson & Peterson, 1981, for review).

Nevertheless, alterations in tumour blood flow have recently been used to exploit the properties of certain therapeutic regimens. For example, Chaplin (1986) has found that 5-hydroxytryptamine (5HT) can enhance the effectiveness of the hypoxic cytotoxin, RSU 1069. Similarly Knapp *et al.* (1986) have used 5HT to enhance tumour response to hyperthermia. In both cases presumably 5HT is acting by decreasing blood flow to the tumour. In contrast, two other vasoactive agents, the calcium antagonists verapamil and flunarizine have been shown to increase tumour blood flow in experimental animals (Kaelin *et al.*, 1982, 1984). This property could therefore be exploited in radiation therapy, where enhanced tumour oxygenation brought about by the increase in tumour blood flow is advantageous.

Flunarizine is a unique calcium antagonist in that its vasodilating properties are seen in the peripheral tissues at concentrations which have little effect on the heart or major blood vessels (Nakayama & Kasuya, 1980). For this reason, together with the findings of Kaelin *et al.* (1984) flunarizine, and a closely related compound, cinnarizine were studied for their ability to radiosensitise two murine tumours, the RIF-1 sarcoma and the SCCVII/St carcinoma.

### Materials and methods

### *Mice and tumour systems*

The tumour lines used in the experiments were the RIF-1 sarcoma and the SCCVII/St carcinoma. The standard protocol for maintenance of the RIF-1 line (Twentyman *et al.*, 1980) was also applied to SCCVII/St. Tumours were implanted on the backs of 12–14 week old C3H/Km female mice by intradermal injection of $2\times10^5$ cells in 0.05 ml Waymouth's medium with 15% foetal calf serum. Tumours were randomly assigned to experimental groups 12–14 days later when tumours were 200–600 mg in weight. The haematocrits of all mice were taken prior to each experiment, by removing 5 $\mu$l blood from the tail, into a capillary tube. The samples were spun in a microhaematocrit centrifuge (Adam's Autocrit, New York) and the value read from a microhaematocrit reader. Mice with haematocrits below 40% were excluded from the experiment.

### *Vasoactive compounds*

Cinnarizine, (1-(Diphenylmethyl)-4-(3-phenyl-2-propenyl) piperazine) and flunarizine, (1-[Bis(4-fluorophenyl)methyl]-4-(3-phenyl-2-propenyl)piperazine) were kindly supplied by Janssen Pharmaceuticals, Beerse, Belgium.

The compounds were prepared for injection immediately before use by suspension in peanut oil (Sigma Chemical Co., St. Louis, Mo), and injected i.p. at $0.01\,ml\,g^{-1}$ mouse weight.

### *Irradiations and assay for tumour response*

Mice were exposed to a single whole body dose of 250 KVp X-rays at a dose rate of $2.85\,Gy\,min^{-1}$ while breathing air.

Tumour radiosensitivity was determined by the *in vivo/in vitro* assay method. Mice were killed by cervical dislocation 18–24 h after irradiation. The tumour was removed, weighed and finely chopped with scissors, then disaggregated into a single cell suspension as previously described (Hirst *et al.*, 1982). The cell number was determined using a haemacytometer, and the cell suspension was diluted and plated at the required cell concentration in plastic tissue culture dishes (Becton Dickinson Labware, Oxnard, Ca) with Waymouth's medium plus 15% foetal calf serum. Two cell concentrations with three dishes per concentration were prepared for each tumour. Dishes were incubated for 12–14 days at 37°C in humidified 5% $CO_2$ in air, after which time colonies with more than 50 cells were scored and used to calculate the plating efficiency and the surviving fraction.

Plating efficiencies of both tumour lines were 15–30%.

### *Normal tissue assay*

The response of a normal tissue to the experimental treatment was determined using a total white cell count after

Correspondence: P.J. Wood.
Received 20 April 1988; and in revised form 21 July 1988.

whole body irradiation. Blood was collected from the mouse tail at different times after irradiation and diluted 1 in 20 in 2% acetic acid using a standard white cell diluting pipette (Pfeiffer Glass Inc.). The white cell count per $mm^3$ was determined using a haemacytometer.

### Results

The time course of the effect of $50\,mg\,kg^{-1}$ cinnarizine injected i.p. on the response of RIF-1 or SCCVII/St tumours to 20 Gy of X-rays *in vivo* was determined and the results are given in Figure 1. Clearly, at the dose given, cinnarizine did not produce a significant increase in cell killing over that for X-rays alone in the RIF-1 tumour. However, this agent produced a significant increase in cell killing ($P<0.05$) in the SCCVII/St tumour when given 2 or 6 h before irradiation.

Figure 2 gives the time course for the tumour responses to flunarizine under the same experimental conditions. In this case flunarizine produced a small enhancement in cell killing in the RIF-1 tumour when given 4 h before irradiation, and a large effect in the SCCVII/St tumour, where maximal sensitisation, a 10-fold increase in cell killing, occurred when the compound was given 45 min before irradiation. These data indicate that at the concentration tested, flunarizine was a better sensitiser than cinnarizine and was more effective in the SCCVII/St than in the RIF-1 tumour. For these reasons, subsequent experiments were carried out using flunarizine in the SCCVII/St tumour.

The response of the SCCVII/St tumour to 20 Gy X-rays with varying doses of flunarizine injected 45 min prior to irradiation was then determined and the results are given in Figure 3. There was sensitisation over a wide range of flunarizine concentrations, with the suggestion of a maximum at $\sim 5\,mg\,kg^{-1}$. Also of interest is that doses of $50–500\,mg\,kg^{-1}$ were slightly less effective than $5\,mg\,kg^{-1}$ flunarizine. The mice showed no signs of toxicity at these higher doses. Thus the most effective concentration of flunarizine to sensitise to 20 Gy X-rays was $\sim 5\,mg\,kg^{-1}$.

Figure 4 gives X-ray dose response curves for the SCCVII/St tumour alone and with $5\,mg\,kg^{-1}$ flunarizine administered 45 min prior to irradiation.

Flunarizine sensitised this tumour over the whole X-ray dose range tested, giving a parallel shift in the curve, equivalent to a 5-fold reduction in hypoxic fraction. The magnitude of this effect was similar to that seen by Hill & Stirling (1987) in the KHT sarcoma for a $4\,mg\,kg^{-1}$ dose of flunarizine.

Finally, the effect of flunarizine on an irradiated normal tissue was studied. Mice were exposed to whole body X-ray doses of 2, 4, 6 or 8 Gy, alone or with $5\,mg\,kg^{-1}$ flunarizine injected 45 min prior to irradiation. Total white cell counts were made before irradiation, daily for 12 days after X-rays, and twice weekly after that, up to 36 days. The experiment was terminated at this time as mice had either died, or white

**Figure 1** Time course for the effect of $50\,mg\,kg^{-1}$ cinnarizine injected i.p. on the sensitivity of (a) RIF-1 and (b) SCCVII/St tumours *in vivo* to 20 Gy X-rays. Cross-hatching: survival after 20 Gy X-rays alone. Points are GM ± s.e.

**Figure 2** Time course for the effect of $50\,mg\,kg^{-1}$ flunarizine injected i.p. on the sensitivity of (a) RIF-1 and (b) SCCVII/St tumours *in vivo* to 20 Gy X-rays. Cross-hatching: survival after 20 Gy X-rays alone. Points are GM ± s.e.

**Figure 3** Dose response for the radiosensitisation of the SCCVII/St tumour *in vivo* by flunarizine given i.p. 45 min prior to irradiation. Cross-hatching: survival after 20 Gy X-rays alone. Points are GM ± s.e.

cell counts had returned to normal. The results are given in Figure 5. Flunarizine had no sensitising effect on normal tissue exposed to X-rays, as measured by this assay. In fact, at the lower doses, recovery of the white cell count appeared to be more rapid in the flunarizine treated groups.

### Discussion

The results may be discussed with reference to the relative effectiveness of cinnarizine and flunarizine as radiosensitisers. It is apparent that, at the initial dose of $50\,mg\,kg^{-1}$, flunarizine is a better radiosensitiser than cinnarizine in both tumours. This result is in agreement with data for other therapeutic endpoints, where flunarizine is considered more potent than cinnarizine (Desmedt *et al.*, 1975). However, since flunarizine is active over a large dose range (Figure 3) it may be argued that cinnarizine may have some activity at doses other than $50\,mg\,kg^{-1}$. This was not tested however, since flunarizine was much more potent than cinnarizine at $50\,mg\,kg^{-1}$, and therefore it was considered unlikely that

**Figure 4** X-ray dose response for the SCCVII/St tumour *in vivo* after (○) X-rays alone or (●) X-rays with $5\,mg\,kg^{-1}$ flunarizine given i.p. 45 min prior to irradiation. Points are GM ± s.e.

**Figure 5** Effect of flunarizine on total white cell count of C3H mice after (a) 2 Gy, (b) 4 Gy, (c) 6 Gy or (d) 8 Gy of X-rays. (○) X-rays alone. (●) X-rays with $5\,mg\,kg^{-1}$ flunarizine given i.p. 45 min prior to irradiation. Points are G.M. ± s.e. Each treatment group consisted of 8 mice, except (c) where 4 mice remained after day 15 to end of the experiment, and (D) where 4 mice remained from day 8 to day 10, 3 mice remained from day 10 to day 11 and 2 mice remained on day 12.

cinnarizine would be more effective than flunarizine at any dose.

Another point of interest is that the injection of the compounds immediately prior to irradiation appears to produce a small increase in tumour radioresistance. This effect was also seen when peanut oil was given alone, although this drug vehicle had no significant effect on tumour radiation response at any other time interval (data not shown). This suggests that the trauma associated with the injection can slightly alter the radiation sensitivity of both tumours.

The effectiveness of flunarizine in relation to the tumour under study is also an important point. The two tumours used have differing hypoxic fractions, the RIF-1 tumour having a low hypoxic fraction of ~1%, whereas the SCCVII/St carcinoma has about 15–20% hypoxic cells. It may be simple to argue that the larger drug effects seen in the SCCVII/St tumour were due to the larger hypoxic fraction in this tumour. However, after 20 Gy only hypoxic cells would remain in the tumour, and the ability of flunarizine to sensitise the SCCVII/St tumour to this dose of radiation with little effect on the RIF-1 tumour, suggests the existence of a subpopulation of hypoxic cells within the former tumour which may be targeted by the combined treatment, and which is absent from the RIF-1 tumour.

The response of the SCCVII/St tumour to varying doses of flunarizine gave a very interesting result. There was a very wide dose range over which this compound produced a radiosensitisation, with a maximally effective dose of $\sim 5\,mg\,kg^{-1}$, above which the sensitising effect was reduced. This type of dose response has been suggested by Kaelin *et al.* (1984) in their blood flow studies, and may be related to the sites of action of this compound. In contrast to most vasoactive agents, and other calcium antagonists, flunarizine at low doses acts at peripheral sites, i.e. the small blood vessels, blood cellular components and the CNS, with little cardiac response (Nakayama & Kasuya, 1980). At $5\,mg\,kg^{-1}$ the peripheral responses may therefore predominate, and be responsible for the radiosensitisation seen in this system. As the concentration of flunarizine is increased, activity at sites in the heart and major blood vessels may come into play, producing changes in cardiac output and blood pressure. This may lead to the occurrence of the steal phenomenon, counteracting the peripheral responses and thus reducing the overall radiosensitisation produced by flunarizine.

If the conclusion to be drawn is that flunarizine is producing enhanced radiation cell killing in tumours by increased oxygenation, the question arises as to how this is achieved. While a discussion of the exact mechanism of action of flunarizine is beyond the scope of this report, data from the literature suggest two distinctly different means of producing radiosensitisation. Firstly, flunarizine may act on vascular smooth muscle in a manner which increases tumour blood flow. This effect, demonstrated by Kaelin (1984), together with the increased tumour oxygen content produced by flunarizine (Vaupel, 1987), suggest that the compound is acting upon the so-called acutely hypoxic cells within the tumour, or those whose oxygen supply is perfusion limited. This conclusion is supported by Jirtle (1988), who demonstrated that an i.v. injection of $1\,mg\,kg^{-1}$ flunarizine reduced the fraction of acutely hypoxic cells by 40% in the SMT-2A rat tumour. Therefore, the greater response of the SCCVII/St tumour to flunarizine over that seen in RIF-1, suggests that the SCCVII/St tumour has a higher proportion of acutely hypoxic cells than does RIF-1. This argument is valid, however, only if vascular occlusion leading to acute hypoxia is partial rather than complete, so that improvement in blood flow from drugs can cause increased oxygenation of acutely hypoxic cells. It follows therefore, that these drugs will also increase oxygen supply to the diffusion limited hypoxic cells within the tumour.

Secondly, the action of this agent on blood cell rheology may also be important. Hypoxia is thought to increase red and white cell rigidity, although this is still speculative

(Parker, 1981). One report has shown that reduced red blood cell deformability occurs with increase in tumour size (Cohen, 1979), and clinical studies in patients with peripheral ischaemic diseases have indicated increased blood viscosity (Schmidt-Schonbein & Volger, 1976; Flaming *et al.*, 1979). Flunarizine has been shown to be effective in improving tissue perfusion (Schetz *et al.*, 1978; Flaming *et al.*, 1979), and increasing red blood cell deformability of rigidified red blood cells (DeCree *et al.*, 1979). Thus it may be argued that flunarizine, by preventing hypoxia induced red cell rigidification, may also be improving tumour perfusion.

If flunarizine is to be considered for clinical use as a tumour oxygenator it will be important to establish its effectiveness over a wide range of radiation doses. The data shown in Figure 4 suggest that its mode of action is to reduce the radiobiologically hypoxic fraction, a mechanism which will be effective only as long as hypoxic cells are present.

Another consideration relating to the clinical potential of this compound is its effect on irradiated normal tissue. Flunarizine did not sensitise the bone marrow to radiation (Figure 5), which may be expected if flunarizine increases tissue oxygenation, since most normal tissues are well oxygenated. However, the bone marrow as a normal tissue is not dose limiting, and in the case of poorly oxygenated normal tissue e.g., cartilage (Dische, 1983), the possibility that flunarizine may increase blood flow and radiosensitivity cannot be ruled out, indicating the need for further normal tissue studies.

Finally, if flunarizine is to be of clinical use, it must be effective in fractionated radiation dose regimens. While no evidence is available at this time to indicate how flunarizine may perform under these conditions, data from other workers suggests that increased delivery of oxygen to tumours can be beneficial in fractionated radiotherapy (Suit *et al.*, 1972; Rojas, 1988). One drawback to the clinical use of this agent has been its very long half-life, and the associated side-effects (Van Neuten & Janssen, 1973; Chouza *et al.*, 1986). The effective doses of flunarizine administered to mice in our system are indeed comparable to those given clinically (Staessen, 1977) and problems with toxicity may be overcome simply by reducing the dose.

The potential of flunarizine as a radiation sensitiser is demonstrated by this study, and this compound is worthy of further investigation.

The authors thank Mr D. Menke and Ms N. Kaul for their excellent technical assistance. This study was supported by grant number CA-25990 from the National Cancer Institute.

### References

CATER, D.B., GRIGSON, C.M.B. & WATKINSON, D.A. (1962). Changes in oxygen tension in tumors induced by vasoconstrictor and vasodilator drugs. *Acta Radiol.*, **58**, 401.

CHAPLIN, D.J. (1986). Potentiation of RSU-1069 tumour cytotoxicity by 5-hydroxytryptamine (5HT). *Br. J. Cancer*, **54**, 727.

CHOUZA, C., CAAMANO, J.L., ALJANATI, R., SCARAMELLI, A., DE MEDINA, O. & ROMERO, S. (1986). Parkinsonian, tardive dyskinesia, akathesia, and depression induced by flunarizine. *Lancet*, **i**, 1303.

COHEN, M.H. (1979). Impairment of red blood cell deformability by tumor growth. *J. Natl Cancer Inst*, **63**, 525.

DE CREE, J., DE COCK, W., GUEKENS, H., DE CLERCK, F., BEERENS, M. & VERHAEGEN, H. (1979). The rheological effects of cinnarizine and flunarizine in normal and pathological conditions. *Angiology*, **30**, 505.

DESMEDT, L.K.C., NIEMEGEERS, C.J.E. & JANSSEN, P.A.J. (1975). Anticonvulsive properties of cinnarizine and flunarizine in rats and mice. *Arzneim. Forsch.* (*Drug Res.*), **25**, 1408.

DISCHE, S. (1983). The clinical use of hyperbaric oxygen and chemical hypoxic cell sensitizers. In *The Biological Basis of Radiotherapy*, Steel *et al.* (eds), Elsevier, 225.

FLAMENG, W., VERHEYEN, F., BORGERS, M. DE CLERCK, F. & BRUGMANS, J. (1979). The effect of flunarizine treatment on human red blood cells. *Angiology*, **30**, 516.

HILL, R.P. & STIRLING, D. (1987). Oxygen delivery and tumour response. Radiation Research. (*Proc. 8th Int. Cong. Radiat. Res.* Fielden *et al.* (eds), **2**, 725.

HIRST, D.G., BROWN, J.M. & HAZELHURST, J.L. (1982). Enhancement of CCNU cytotoxicity by misonidazole: studies of possible therapeutic gain. *Br. J. Cancer* **46**, 109.

JIRTLE, R.L. (1988). Chemical modification of tumour blood flow. *Int. J. Hyperthermia*, **4**, 355.

JIRTLE, R., CLIFTON, K.H. & RANKIN, J.H.G. (1978). Effects of several vasoactive drugs on the vascular resistance of MT-W9B tumors in W/Fu rats. *Cancer Res.*, **38**, 2385.

KAELIN, W.G., SHRIVASTAV, S., SHAND, D.G. & JIRTLE, R.L. (1982). Effect of verapamil on malignant tissue blood flow in SMT-2A tumor-bearing rats. *Cancer Res.*, **42**, 3944.

KAELIN, W.G., SHRIVASTAV, S. & JIRTLE, R.L. (1984). Blood flow to primary tumors and lymph node metastases in SMT-2A tumor-bearing rats following intravenous flunarizine. *Cancer Res.*, **44**, 896.

KNAPP, W.H., DEBATIN, J., LAYER, K. & 4 others (1985). Selective drug-induced reduction of blood flow in tumor transplants. *Int. J. Radiat. Oncol. Biol. Phys.*, **11**, 1357.

KRUUV, J.A., INCH, W.R. & MCCREDIE, J.A. (1967). Blood flow and oxygenation of tumors in mice II. Effects of vasodilator drugs. *Cancer*, **20**, 60.

MATTSSON, J. & PETERSON, H.I. (1981). Influence of vasoactive drugs on tumor blood flow (review). *Anticancer Res.*, **1**, 59.

NAKAYAMA, K. & KASUYA, Y. (1980). Selective abolition of Ca-dependent responses of smooth and cardiac muscles by flunarizine. *Japan. J. Pharmacol.*, **30**, 731.

PALLAVICINI, M.G. & HILL, R.P. (1983). Effect of tumor blood flow manipulations on radiation response. *Int. J. Radiat. Oncol. Biol. Phys.*, **9**, 1321.

PARKER, J.C. (1981). Effects of drugs on calcium-related phenomena in red blood cells. *Fed. Proc.*, **40**, 2872.

ROJAS, A. (1988). Oxygen radiosensitisation: a clinical reality or a mirage? *Br. J. Radiol.* (in press).

SCHETZ, J., BOSTOEN, H., CLEMENT, D. & 4 others (1978). Flunarizine in chronic peripheral arterial disease: A placebo-controlled, double-blind, randomized multicentre trial. *Curr. Ther. Res.*, **23**, 121.

SCHMIDT-SCHONBEIN, H. & VOLGER, E. (1976). Red-cell aggregation and red-cell deformability in diabetes. *Diabetes*, **25** (suppl. 2), 897.

STAESSEN, A.J. (1977). Treatment of circulatory disturbances with flunarizine and cinnarizine. *VASA*, **6**, 59.

SUIT, H.D., MARSHALL. N. & WOERNER, D. (1972). Oxygen, oxygen plus carbon dioxide and radiation therapy of a mouse mammary carcinoma. *Cancer*, **30**, 1154.

TWENTYMAN, P.R., BROWN, J.M., GRAY, J.W., FRANKO, A.J., SCOLES, M.A. & KALLMAN, R.F. (1980). A new mouse tumor model system (RIF-1) for comparison of end-point studies. *J. Natl Cancer Inst.*, **64**, 595.

VAN NEUTEN, J.M. & JANSSEN, P.A.J. (1973). Comparative study of flunarizine and cinnarizine on smooth muscle and cardiac tissues. *Arch. Int. Pharmacodyn. Ther.*, **204**, 37.

VAUPEL, P. & MENKE, H. (1987). Blood flow, vascular resistance and oxygen availability in malignant tumours upon intravenous flunarizine. *Adv. Exp. Med. Biol.*, **215**, 393.